# ‘We don’t have the right to get sick’: A qualitative study of gaps in public health insurance among Im/migrant women in British Columbia, Canada

**DOI:** 10.1371/journal.pgph.0001131

**Published:** 2023-01-26

**Authors:** Shira M. Goldenberg, Shaina Schafers, Maggie Hamel-Smith Grassby, Stefanie Machado, Ruth Lavergne, Mei-ling Wiedmeyer, on behalf of the IRIS Study Team

**Affiliations:** 1 Division of Epidemiology and Biostatistics, San Diego State University, San Diego, CA, United States of America; 2 Centre for Gender and Sexual Health Equity, St. Paul’s Hospital, Vancouver, BC, Canada; 3 Faculty of Health Sciences, Simon Fraser University, Burnaby, BC, Canada; 4 Department of Medicine, University of British Columbia, St. Paul’s Hospital, Vancouver, BC, Canada; 5 Department of Family Medicine, Dalhousie University, Halifax, NS, Canada; 6 Department of Family Practice, University of British Columbia, University Boulevard, Vancouver, BC, Canada; PLOS: Public Library of Science, UNITED STATES

## Abstract

Globally, the exclusion of im/migrants from public health care systems remains a significant health and human rights issue, calling into question claims of ‘universality’ of public health systems where eligibility for coverage is determined by immigration status. We aimed to describe lived experiences of health insurance coverage and the health and social impacts of gaps in health insurance amongst im/migrant women in British Columbia (BC), Canada. This analysis draws on qualitative in-depth interviews (n = 78) with im/migrant women and im/migrant-focused service providers (n = 10) conducted between July 2018-March 2021 in Metro Vancouver, BC, as part of a larger community-based, mixed-methods study of im/migrants’ health access (IRIS). In contrast to common perceptions of Canada’s health system as ‘universal’, participants faced multifaceted barriers to health insurance and necessary healthcare for themselves and their families following arrival in BC. Narratives highlighted the ways in which ineligibility for public health insurance coverage resulted in unmet needs for essential sexual and reproductive health and preventive care among im/migrant women, children, and families. Participants also described ineligibility for public health insurance as resulting in a high economic burden, and that exclusion from public health insurance perpetuated experiences of discrimination, invisibility, and exclusion from systems of care amongst im/migrant participants. Despite these structural challenges, participant narratives highlighted the crucial role of community-based supports for minimizing harm and navigating oppressive immigration and health systems. Changes to immigration and health policies are required to remove barriers to public health care for im/migrant women and ensure that Canada’s health system is accessible to all. Expanding health insurance options to cover all residents and decoupling health insurance eligibility from immigration status are recommended, alongside implementation of ‘Sanctuary’ policies at the local level.

## Introduction

Globally, the exclusion of im/migrants (the term “im/migrant” includes the diversity of refugee, immigrant and migrant people born in other countries who entered Canada, including long-term and recent arrivals, asylum seekers, economic and undocumented im/migrants [1]) from public health care systems remains a significant health and human rights issue, calling into question claims of ‘universality’ of health systems where eligibility for coverage is determined by immigration status. Although the stark health inequities faced by im/migrants are longstanding [2–5], public health evidence that has emerged during the COVID-19 pandemic has illuminated the substantial barriers to health care faced by im/migrants globally [6,7]. Previous research describing health access inequities faced by im/migrants has pointed to lack of sufficient health insurance coverage [8,9], which has been a subject of increasing attention during COVID-19 [10].

While access to care for im/migrants has often been studied through the lens of behavioural science or health services, growing evidence suggests the need to consider the ways in which immigration and health policies shape access to care and related outcomes. In 2021, im/migrants across destination contexts continue to report egregious gaps in healthcare access [6,7,11–13], highlighting the urgent need for program and policy changes to close these gaps. The ‘right to health’–that is, framing health as a human right, legal entitlement and fundamental matter of social justice–has long been established in international human rights law, as are principles of equality and non-discrimination [14]. This principle was first described in the 1946 WHO constitution, subsequently also mentioned in the 1948 Universal Declaration of Human Rights, and now included in the Sustainable Development Goals.

As affirmed by the UN General Assembly resolution in 2019, universal health care is defined as a state in which “*all people have access*, *without discrimination*, *to nationally determined sets of the needed promotive*, *preventive*, *curative*, *rehabilitative and palliative essential health services*, *and essential*, *safe*, *affordable*, *effective and quality medicines and vaccines*, *while ensuring that the use of these services does not expose the users to financial hardship*, *with a special emphasis on the poor*, *vulnerable and marginalized segments of the population*” [15]. Additionally, universal health care has been identified as a crucial priority in the United Nations’ Sustainable Development Goals *(Target 3*.*8)* [16]. Most nation states, including Canada, have signed such global and national policy instruments endorsing universal health care, yet few have sufficiently prioritized address the ‘right to health,’ for migrants [17], and in many cases combinations of immigration and health policies explicitly exclude certain groups. For example, few settings have implemented sufficient policy and programmatic changes to fully realize the ‘right to health’ as it relates to healthcare access for im/migrants with precarious status–that is, immigration status marked by the absence of rights and entitlements normally associated with permanent residence or citizenship [18]. This includes those with certain temporary statuses (e.g., work permit), undocumented individuals, those experiencing status changes, and those with claims in process (e.g., refugee claimants) [8,19].

Ranking among larger global economies, Canada relies heavily on im/migrant labour and support and is often portrayed as a multicultural setting characterized by universal healthcare [20]. Canada has signed several relevant global commitments, including the UN Global Compact for Migration [21] that commits to providing care and assistance to im/migrants. While not legally binding, such commitments represent shared values and provide opportunities to guide dialogue towards policy reform. Despite these commitments and purported values, the concept of universality for residents in the *Canada Health Act*–the primary legislation underpinning Canada’s public health insurance system–continues to hold a very narrow definition, including only medically necessary hospital and physician services for residents, with eligibility varying by jurisdiction [22].

Although a growing body of research has documented the negative health, economic, and social consequences of a lack of health insurance among undocumented, Latinx, and low-income communities in other high-income countries such as the U.S. [8,10] few recent empirical studies have described im/migrants’ experiences with health insurance coverage in purportedly ‘universal’ health system contexts such as British Columbia, Canada [23,24]. Over the past decade, most research on gaps in health insurance and its implications among undocumented migrants has been conducted in the U.S., and research conducted in Canada has primarily addressed insurance issues and gaps faced by refugees and refugee claimants [19,25,26]. Given the limited focus of prior research on the experiences of broader populations of im/migrants in Canada, such as undocumented migrants and others with precarious status, our aim was to describe the lived experiences of eligibility for public health insurance coverage and the health and social impacts of gaps in health insurance among im/migrant women and providers in British Columbia (BC), Canada.

## Methods

The Evaluating **I**nequities in **R**efugee and **I**mmigrant’s Health **S**ervices Access (IRIS) study is a five-year, mixed-methods research collaboration between academics, community members, and local im/migrant support and advocacy organizations in BC. IRIS data collection activities include qualitative interviews as well as a quantitative arm involving analyses of large, population-based administrative data from health and immigration systems. IRIS is guided by a community engagement framework that draws on principles of community-based research [27], including iterative community collaboration across all stages of question development, data collection and analysis. This also includes a commitment to community advocacy through co-creation of knowledge dissemination products and knowledge translation and exchange with participants, community partners and the broader im/migrant community, including through several multilingual community advisory boards (English, Dari, Farsi, Spanish, Tigrinya). As described previously [28], research priorities, questions, and recruitment strategies were informed by formative consultations with community partner agencies and im/migrant women.

### Data collection

#### Setting

This study was conducted in Metro Vancouver, BC. BC is among Canada’s top destination provinces for im/migrants, where >25% of the population are im/migrants, with >40,000 arrivals annually.^10^ As in other provinces, urban areas such as Metro Vancouver attract the majority of newcomers.^11,12^ In BC, Canadian citizens and immigrants with permanent residency status who are considered BC residents are eligible for health insurance under BC’s **Medical Services Plan (MSP).** Some people who hold temporary status (e.g. study or work permits) valid for a period of six or more months may also be deemed residents, and are therefore eligible [29]. MSP requires that newly eligible individuals (i.e., new Canadian residents or individuals moving from a different province) wait three months prior to obtaining health insurance coverage. Previous research from BC has shown the ways in which this waiting period results in mistrust, stigma, and negative health consequences among immigrant women [28]. Additionally, certain groups of migrants, such as refugees and refugee claimants, receive federal coverage under an alternative health plan *(Interim Federal Health Plan*, *IFHP*), which provides basic and supplemental insurance only until individuals become eligible for provincial health insurance such as MSP. People with other forms of temporary status or no status (including expired temporary permits) do not qualify as residents of BC under the current policy, and are therefore not eligible for MSP. Visitors and people impacted by the 3-month wait (including those moving to BC from another province) are instructed to purchase private insurance or temporarily retain their prior insurance; however, private plans are typically limited in covering only emergency care, and exclude pre-existing conditions, including pregnancy.

#### Participants

This community-based qualitative study drew on in-depth, one-on-one interviews with im/migrant women (N = 78) and im/migrant-focused service providers (N = 10) conducted from July 2018 to March 2021. *Eligibility criteria* for im/migrant women were self-identifying as a woman (cis or trans); aged 18–49; moved to Canada from another country in the last 5 years; and able to provide informed consent. *Eligibility criteria* for service providers were individuals employed in the health, social, or legal sectors with im/migrant women (e.g., clinicians, staff at community-based organizations) and able to provide informed consent. Sampling was led and carried out iteratively by research team members in collaboration with community-based organizations and networks across Metro Vancouver. Participants were *purposively selected* [30] to include a diversity of perspectives and experiences, including those related to place of origin, im/migration status, language, and age, in close collaboration and with the support of community partners. Although there were no exclusion criteria based on immigration status, sampling prioritized the perspectives of those with more underrepresented and precarious immigration statuses–including undocumented, temporary work permit, refugee claimant, or recent refugees [31]. Service providers were selected to cover a range of im/migrant and health service roles, including healthcare providers and social services (eg., outreach, settlement). Recruitment was supported by community-based programmes, study posters, and outreach to community-based organizations. Interview sampling and data analyses were carried out iteratively.

#### Interview procedures

Interviews were conducted in the language in which participants were most comfortable speaking (either Spanish, English, Farsi, Dari, or Tigrinya) by trained multilingual and multicultural interviewers with lived migration experience. Interviewers received training on qualitative interviewing, confidentiality, and data management protocols. Questions were open-ended and addressed im/migration history; experiences with sexual and reproductive health, other types of care, and social services in Canada; and recommendations for improving health service access. Interviewees provided written informed consent prior to participation and confidentiality protocols were followed. All sessions were conducted in each participant’s preferred language, audiotaped, and lasted approximately 1.5 hours. A brief demographic questionnaire was administered to describe characteristics of the qualitative sample. Follow up interviews were conducted with a subset of im/migrant women (n = 14) who were invited to provide feedback on preliminary findings and expand on prior interview themes in greater depth. Participants who expressed particularly strong and diverse perspectives and were interested in a follow-up interview to expand on previous themes were selected for follow-up interviews. Im/migrant women received an honorarium of $40 CAD and received referrals to community-based social, health, and legal supports as needed. Interviews with service providers elicited their professional experiences working with im/migrant women, gaps in services, and perspectives on how to improve im/migrant women’s health and wellbeing.

#### Ethical considerations

IRIS is approved through the Simon Fraser University (SFU) and Providence Health Care/University of British Columbia (UBC) harmonized research ethics boards. During the informed consent process, participants received detailed information regarding the purpose of the study, the voluntary and confidential nature of participation, and its risks and benefits, and provided written informed consent prior to enrolment. Rigorous confidentiality and data security and management procedures were followed.

### Data analysis

Interviews were transcribed, translated and accuracy-checked. All personal identifiers were removed. Data were inductively coded [32] by a coding team of trained community researchers, trainees, and coordinators, most of whom had lived in/migration experience. Coding was managed using NVivo v12 (QSR, AUS) and the team maintained a detailed study codebook. Data analysis drew on an inductive content analysis approach. We initially used content analysis to generate a set of initial codes to organize the data and describe key features. As interviews were conducted, the coding team iteratively adapted the coding scheme using analysis of initial transcripts and collaborated to establish a shared understanding of codes and their definitions. The second stage of analysis involved the first three authors reviewing codes pertaining to access to health services for women, focusing on emerging themes related to health insurance coverage. As the emergent analysis revealed that participants’ narratives frequently used the language and framing of ‘rights’ to critique and describe their experience with BC’s health insurance and services, during the final stage of analysis we drew on conceptualizations of the ‘right to health’ [14] to analyze participants’ perspectives on health insurance coverage, as well as its health and social impacts (e.g., unmet needs for care, discrimination, cost). Preliminary results were shared with im/migrant women participants and community partners (e.g., community advisory board meetings, partner meetings, and virtual sharing of plain-language materials and infographics), who were invited to provide feedback on the findings and their perceived accuracy, relevance, and implications to inform data interpretation as it progressed.

## Results

### Participant characteristics

Among 78 im/migrant women participants, the median age was 31 and the median duration since arrival in BC was 1.59 years. Primary regions of origin were Latin America and the Caribbean (Table 1), followed by the Eastern Mediterranean and Africa. Three-quarters of participants spoke Spanish, with 21.8% (n = 17) speaking Farsi, Dari, and/or Turkish and 9% speaking Tigrinya, Amharic, and/or Arabic. The majority had experienced multiple im/migration status changes since arriving to BC/Canada; at the time of interview, 37.2% of participants were either a permanent resident or citizen, while the remainder had more precarious immigration status (e.g., undocumented, work or student permit, tourist, or refugee claimant/asylum applicant). Providers had a range of professional roles and represented various places of origin and languages spoken (Table 2).

**Table 1:** Im/migrant women: Participant characteristics (N = 78).

Variable	n (%)
Age, *years*^a^	31 (18–49)
**Region of origin**	
• Region of the Americas and Europe (Colombia, Dominican Republic, Guatemala, Chile, Panama, Honduras, Spain, Mexico, Venezuela)	59 (75.6%)
• Eastern Mediterranean and African Regions (Afghanistan, Iran, Syria, Eritrea, Ethiopia)	19 (24.4%)
**Languages Spoken** ^**b**^	
• Spanish	75.6%)
• Tigrinya/Amharic/ Arabic	(9.0%)
• Farsi/ Dari/ Turkish	21.8%)
• Hindi/ Urdu	3 (3.8%)
**Immigration Status at Time of Interview**	
• Permanent Resident/ Citizen	37.2%)
• No Status / Unknown	29.5%)
• Work / Student Permit	(11.5%)
• Refugee Claimant / Asylum Applicant	(10.3%)
• Tourist Visa	9 (11.5%)
Time in BC/Canada, *year*^a^	1.59 (0.16-11)

^a^Median (range).

^b^Many participants spoke more than one language; interviews were conducted in the language they felt most comfortable speaking.

NOTE: Data are N (#) of participants, unless otherwise indicated.

**Table 2:** Service providers: Participant characteristics (N = 10).

Variable	
Age, *years*^a^	43 (33–57)
Gender	
• Woman	10
• Man	0
• Non-binary/Other	0
Place of birth	
• Canada	3
• Other (e.g., Asia, UK, Africa)	6
• Prefer not to say	1
Years working with im/migrant women, *years*^a^	7 (2–30)
Roles	
• Community advocate/outreach worker	2
• Nurse/Nurse Practitioners	4
• Settlement worker	2
• Other (e.g., social worker)	2

^a^Median (range).

NOTE: Data are N (#) of participants, unless otherwise indicated.

### Findings

Following arrival to BC, im/migrant women commonly faced ongoing and severe barriers to health insurance coverage and health care—in stark contrast to common discourse framing Canada’s health system as providing access to health care, regardless of ability to pay, Our analysis identified the following five themes which describe various dimensions of im/migrant women’s access to and experiences with public health insurance coverage: 1) ***Ineligibility for public health insurance***–which in BC is tied to immigration status and requires a mandatory waiting period following arrival (3 months)—***resulted in serious negative impacts*** related to unmet needs for sexual and reproductive health, preventative, and curative services, and negative health consequences. 2) Women for whom health services were covered by insurance (e.g., women without insurance and in some cases, those requiring care not covered by MSP) described the ***economic burden of care*** to be unaffordable, unpredictable, and deeply stressful for families. 3) While ***becoming eligible for public health insurance*** (typically through immigration status changes) often improved access to care, women often received confusing, contradictory and incomplete information about health insurance that led to gaps in care for those affected by status changes. 4) Ineligibility for health insurance and the ***exclusion from systems of care*** this engendered was perceived as a form of ***discrimination*** amongst im/migrant women. 5) In response to these structural barriers, narratives highlighted the crucial role of ***community-based supports*** for minimizing harm and navigating exclusionary immigration and health systems. Below, we expand on these themes.

### 1) “A migrant doesn’t receive benefits”: Ineligibility for public health insurance and unmet healthcare needs

Of the 78 im/migrant women, almost three-quarters (n = 57) arrived in Canada without permanent residency and as a result experienced varying periods of precarious im/migration status that would have impacted eligibility for public health insurance in BC. For example, arriving as a visitor to Canada, changes in employment resulted in status gaps, reliance on employer or school for status, or periods of no status after rejected status claims. The remainder of the sample experienced some privileges associated with landed permanent residency, but experienced discrimination at the point of healthcare access (see theme 4—*Discrimination*, *invisibility*, *and exclusion from care*), and specific gaps depending on provincial regulations such as the three month wait period [28].

Participants overwhelmingly described the ways in which exclusion from government-funded public health insurance contrasted with their expectations when coming to Canada, which some identified as an infringement of their basic rights:

*They treat us like…we don’t have rights…The most important thing in a family is to feel healthy, to feel well, and we don’t even have that (Latin American woman*, *3 years in BC)*

Such policy structures that failed to address the realities of im/migrants with precarious status in BC were often seen as placing im/migrant women and families in a state of limbo between their places of origin and destination–ineligible for coverage and unable to access affordable care in either setting.

As a result of being ineligible for public health insurance, im/migrant women faced high unmet needs for hospital, specialist, and primary care services. These included sexual and reproductive health (SRH) services such as contraception, STI testing, prenatal care, labor and delivery care, preventive check-ups, and treatment for ongoing and emergency conditions. Particularly severe impacts were faced by pregnant women, who were often left completely excluded from coverage, since pregnancy is considered a pre-existing condition for which private insurance companies will not provide coverage. As a result, participants faced gaps in prenatal care. Many did not receive standard-of-care (e.g., early and regular prenatal visits), with numerous women reporting their first prenatal visits as late as six or seven months into pregnancy–a violation of both maternal and child health rights [14]. These concerns were particularly alarming given that participants’ experiences of pregnancy were often associated with complex, traumatic health needs or obstetric emergencies, thus adding financial stress at an already-stressful moment:

*In the moment when your labor is not going as planned*, *in addition to that emotional pain that you feel when you realize that your labor is not going to be as you wanted, you have the worry about the fact that you know that everything is not going to cost $1000 (CAD), you know that it is going to be around $10,000 (CAD). I don’t know what hurts more. (Service provider)*

As a result of policies excluding them from public health insurance coverage, women often described feeling powerless to protect the health of themselves and their families and expressed desperation that no one would fall ill; such concerns were compounded by the gendered nature of caregiving. Participants commonly described immense concern about the cost and burden of falling ill while being uninsured. Those who had only dealt with minor health issues since arriving expressed profound relief, recognizing that they would not have been able to access care had they faced more complex needs. As one participant explained:

*When I felt that I was beginning to get sick I would tell myself that I didn’t have a right to get sick, I would say “shake it off*, *you can’t get sick”…thank goodness nothing else happened to me. When the hospital bill arrived I said, “no, I won’t get anything else”. (Latin American woman, 5 years in BC)*

### 2) “For me, that’s a fortune”: Prohibitive costs of care and economic burden

High out-of-pocket costs for healthcare not covered by BC’s public health insurance program posed a prohibitive barrier to care that shaped access to care and types of care accessed, and in some cases resulted in foregone care altogether. High and unanticipated costs represented an extreme economic burden and source of stress for families. In many cases, accessing care meant forgoing other essential needs (e.g., food, housing); in cases where families could not pay their bill, some reported having collection agencies come after them after giving birth in a hospital.

Many participants described concern regarding a lack of clear information provided to im/migrant women and families regarding what the cost of care would be until arrival at the point of care, or in some cases, after services were delivered. Clinics and physicians in BC are allowed, and it is common practice, to charge fees for “uninsured services” that are much higher than the insured rate. Despite the College of Physicians and Surgeons of BC recommending that its’ members consider what is “reasonable” and what a person can afford, there are no established guidelines for routinely covered services.

Cost considerations restricted women’s agency in accessing types and modalities of care related to SRH care (e.g., prenatal care, labor and delivery, contraception, ultrasounds) and resulted in unmet or delayed access to preventative check-ups and lab visits. For example, some participants noted that they would have rather given birth in the hospital, but opted for homebirth to avoid prohibitive costs. For participants who delivered in-hospital, high hospital fees resulted in pressure to leave quickly, sometimes in poor condition, and in many cases earlier than they felt was appropriate. One woman paid $15,500 [CAD] for a caesarian delivery and left hospital before full recovery to avoid additional costs. Another uninsured woman described multiple instances during which she was advised to go to the hospital during her pregnancy–because she began to bleed while pregnant–but left after being informed of the prohibitive costs:

*I went to emergency and just for going into the hospital they wanted to charge me $1000 [CAD]*, *and I got nervous and left, because I didn’t have [the money]. With only my husband working, rent, I mean, honestly…(Latin American woman, 4 years in BC)*

In addition to the challenges faced by uninsured women, both insured and uninsured women faced cost-related barriers to services that are not covered by public insurance (e.g., medications). For example, while many participants from Latin America described accessing sexual health screenings or low/no-cost contraception (e.g., oral contraception, surgical) in home countries, upon arrival to Canada, these services were prohibitively expensive for many. While some provinces do cover select prescription medications for individuals who are already enrolled in public health insurance—requiring that many Canadian citizens hold private health insurance for any non-covered prescription medications—barriers were greatly exacerbated for uninsured im/migrants. This was largely due to the two-step process to accessing prescriptions necessitated by a gatekeeper model that requires patients to visit a primary care provider for diagnosis and prescription, which must then be filled and paid for at a pharmacy, with added costs at each step for those without insurance. As a result, contraceptive health care choices were restricted, and many women and their partners were forced to use whichever method was offered for free or forgo contraception entirely. These logistical barriers and challenges caused confusion and anguish for participants, particularly for those used to healthcare all being under one umbrella in home countries.

### 3) “It was an improvement for us”: Impacts of becoming eligible for health insurance

For women who recently arrived to BC or experienced periods of precarious status, becoming eligible for public health insurance often dramatically improved access to care. This was typically achieved following immigration status changes or the end of the mandatory waiting policy after moving to BC. Due to the stress and costs associated with being uninsured, becoming eligible for public health insurance was accompanied by peace of mind and improved access for women and their families, including formerly uninsured children:

*We did the papers for our residency and they gave my husband a work permit*. *Immediately we got insurance for my son so that he could have access to healthcare. (Latin American woman, 3.5 years in BC)*

*It was an improvement for us…here, without being legal it’s hard…with immigration status it’s different*, *and you can access several supports. (Latin American woman, 1.17 years in BC)*

Despite the potential for status changes to improve access to healthcare coverage, women and service providers reported confusing, contradictory and incomplete information about health insurance that led to gaps in care for those affected by status changes. Those newly eligible for public health insurance described facing additional confusion and barriers associated with the 3-month waiting period for health insurance coverage [28,33]. For example, one woman did not receive any prenatal visits during the wait period, because she was turned away for not having proof of insurance and did not know she could pay out-of-pocket. She later miscarried and does not know the cause. Others thought they could access care during this wait period—since there was no mention of cost at the point of care—but were later sent unexpected bills.

Additionally, there have been changes to Canada’s Interim Federal Health Program (IFHP), which grants temporary health insurance and other social benefits to refugees and refugee claimants [34]. Both im/migrant women and providers found the administrative hurdles and changes to the IFHP to be confusing and difficult to navigate; for example, knowledge of coverage and specific services covered, cost, billing procedures, and acceptance of IFHP by different providers were all described as unclear and challenging to navigate. Whereas refugee women largely expressed concern that they felt ‘in the dark’ regarding how, where, and when they could use IFHP and the implications of being unable to effectively access care under IFHP for themselves and their families, providers focused more on the administrative challenges this posed, expressing concern about the impact this had on their clients.

*It wasn’t until my boy got sick that the lady from the Church told me that with that paper [IFHP] they were not going to charge me anything and they will take care of my son*. *But I didn’t know the paper was useful for everything. I didn’t know. (Latin American woman, 1 year in BC)*

*They [providers] need to know*, *OK, this person has federal coverage, and then [patients] need to know which providers they can go to, including obstetricians that they might need, that are registered under Blue Cross, Federal Health, so that they don’t get billed, so it’s…yeah. This confuses me, and I’ve been doing this for seven years, so how do folks know this? It’s really, really difficult. Lots of barriers. (Service provider)*

These challenges often persisted long-term, with some women recalling that they did not realize they were eligible for health insurance and therefore did not seek out health services, even long past the point where they first became technically eligible for health insurance coverage.

*I don’t think we had a family doctor until like two years that we were in Canada…Cause nobody really would*, *like- I think they charge for it before, no?…Can you get a family doctor if you don’t have, your, like PR or citizenship or something? (Latin American woman, 11 years in BC)*

### 4) “They will leave you to die? Because you’re not from here?”: Discrimination, invisibility, and exclusion from care

The multiple ways in which im/migrant women and their families were excluded from care resulted in feeling ‘invisible’. Women struggled to identify where they could access services “*as a human being here*, *regardless of status”* (Latin American woman, 1 year in BC). Providers concurred that the needs of im/migrants–particularly those who are uninsured—are infrequently acknowledged in front-line health care settings, and as a result, staff are often unsure and under-resourced to appropriately support these patients.

Experiencing the impacts of structural racism, xenophobia, and gender inequity built into health and immigration systems and policies, experiences of poor treatment or being unable to access services at the point of service delivery were perceived as deeply discriminatory and unjust by im/migrant women:

*[It was] racism. Because for a health service it shouldn’t matter if you’re from…well, I know that obviously not all of us can enjoy the privileges of*, *like, those from here, Canadians, but they also don’t have the right to push you aside. If you get sick, they will leave you to die? Because you’re not from here? (Latin American woman, 4 years in BC)*

The experiences of undocumented women and mixed-status families highlighted the ways in which administrative procedures in clinics and other health service delivery settings–such as asking for proof of health insurance coverage–were often deeply-fear provoking and resulted in avoidance of care. In an alarming number of cases, participants recounted xenophobic experiences in clinics and hospitals that created significant mistrust and fear that accessing care would result in negative immigration consequences:

*If you go to a health centre and they say…”Why are you in this country if you don’t speak English? Why are you here?”…yes*, *going there, I perceived that they would not open their doors and I felt a lot of fear. It was practically that, by asking for medical attention it was a button to be sent home instead of to receive medical attention. (Latin American woman, 6 months in BC)*

*[At the hospital] they started to question us*, *to ask us for papers… what we did was better to get out quickly…we were afraid, I mean, because as soon you get out of the hospital you see a lot of police officers…and when they start questioning you, “let me see your papers”…well, what option is left but to leave… [even though] my hands swell, my face swells…no, I can’t go to the doctor.(Latin American woman, 5 years in BC)*

### 5) Supportive role of community-based organizations

Despite these immense structural inequities, supports provided by community-based organizations and im/migrant-friendly providers who supported them were essential for reducing harm and supporting system navigation. Local community-based, non-profit organizations–particularly those staffed and led by im/migrants—played a crucial role in connecting women to low/no-cost healthcare, and provided essential socio-economic supports (e.g., food, economic assistance). In many cases, these efforts were successful in advocating for reduced or no-cost services (e.g., pregnancy care).

*Related to the pregnancy I cannot complain… [Community Based Organization] helped me a lot*, *a lot, a lot. With the hospital, they treated me well. I was checked by the doctor. For me, thanks to [Community Based Organization]…my baby is doing well. I had a place to give birth… I had no problems related to that. I have not gotten sick; I have not had to go to the hospital. Now, [my baby can] go to the doctor, because [Community Based Organization} helped me get [his medical card], if not, I wouldn’t have been able to. (Latin American woman, 8 months in BC)*

Providers and participants alike spoke of these efforts being under-resourced and under-prioritized by the government. One provider blatantly acknowledged that while there was an official understanding in their healthcare setting that all im/migrants have the right to access care, in practice *“there are still no answers for how to treat people without [public health insurance]*.*”* Another participant discussed the gaps created by the defunding of Vancouver’s only refugee health clinic:

*I had a medical need and I went with my kid to a walk-in clinic where they had told me that they see you without a problem*, *but when I arrived there, it was closed. They closed it because the government stopped the funding for that clinic, so they closed it. (Latin American woman, 5 years in BC)*

Participants discussed the need for increased awareness and protective policies that support the needs of communities who are made invisible by current health and immigration systems. As one recently arrived woman noted, making the experiences of this population more visible and advocating for their needs were described as important steps towards this:

*It is like a hidden community from the government…[the community should] fight more so that the government does not discriminate or…focus so much on the person’s status when offering services (Latin American woman, 6 months in BC)*

## Discussion

This study describes the ways in which ineligibility for public health insurance violates the ‘right to health’ among im/migrant women in Canada. As a result of exclusionary health and immigration policies, participants faced gaps in health insurance eligibility which resulted in barriers to health care and unmet needs for essential and urgent sexual and reproductive health and both curative and preventive care for both im/migrant women and children. Exclusion from care was also perceived to be discriminatory and posed major economic stressors for im/migrant women and families. Findings suggest that the realities of im/migrants with precarious status in destination countries such as Canada are often made invisible through policy structures that place them in a state of limbo between places of origin and destination; this invisibility must be better addressed in all migration destination settings, and particularly in settings which espouse to provide inclusive and universal healthcare.

Although im/migrant communities have been working to call attention to this issue, im/migrants continue to be excluded from public health insurance in Canada and many other settings globally [8,10,19,24,35]. Other scholars have noted the ways in which the restrictive definition of a ‘resident’ compromises the universality of insurance, leaving unacceptable gaps in care for certain groups of im/migrants in Canada [22]. Yet as countries continue moving towards universal health coverage, the obligation to realize the ‘right to health’ for im/migrants–particularly those with precarious status—continues to be under-prioritized. Consistent with research from the U.S. demonstrating gaps in access to care for uninsured people [8,10,35], our findings highlight a range of harms generated by health and immigration policies that exclude certain im/migrants from healthcare, challenging national rhetoric regarding the purported ‘universality’ of Canada’s health system [36]. Building on studies which have identified the roles of both health insurance and immigration status as determinants of health access for im/migrants, our study provides novel insights regarding the ways in which these policies intersect in ways that violate the ‘right to health’ and produce inequity. Additionally, findings show how the failure to provide inclusive access to ‘health for all’ perpetuates structural racism, xenophobia, and health inequity [11,37].

Our findings highlight the need for ***health policy reforms*** that expand access to health insurance for all residents, regardless of immigration status. This could be accomplished by expanding the definition of “resident” in the Canada health act to include all people living in Canada. Canada is a signatory to the *Universal Declaration of Human Rights*, which obliges signatory countries to guarantee accessible health care for all residents and the provision of healthcare based on need is a foundational principle of the *Canada Health Act* [36,38]. Furthermore, a 2018 decision by the United Nations denounced Canada’s health system for discriminating based on immigration status and advised the country to review its health legislation [39]. In BC, such changes can be immediately implemented by the *Medical Services Commission* to decouple health insurance eligibility from immigration status or duration of residence.

Many jurisdictions have found ways to provide health access for im/migrants, regardless of immigration status. In France, Portugal, Spain, and the Netherlands, all im/migrants are eligible for comprehensive access to health services based on proof of identity, location of residence in the country, and evidence of financial need [40]. In France, im/migrants with precarious status, are granted access to the same healthcare services as citizens and permanent residents and processed through a parallel administrative system, which is similar in Spain and Portugal [40]. In the Netherlands, im/migrants with precarious status are eligible for primary care, midwives, dentists, hospital emergency services, nursing homes and other services supported by a unique government fund. Alternatively, Thailand has a voluntary health insurance scheme for migrant workers financed by annual premiums that cover spouses and children [41], which has improved screening and treatment for infectious diseases, increased access to health services, and reduced risk of catastrophic out-of-pocket expenditure [41,42]. Thus, there are multiple options for reforming health systems to improve access to health services for im/migrants with precarious status who are currently either excluded from all forms of public health insurance or are granted differential access to alternative plans (e.g., IFHP). Decoupling health insurance eligibility from immigration status [28], and focusing on the development and implementation of standards or other regulations governing the cost of healthcare that is rendered outside of the context of provincial health insurance are among these. In addition, improving public communication and the provision of clear information regarding insurance coverage, services covered, cost, and processes for newcomers is urgently needed. Many participants spoke about the confusing nature of navigating contradicting information (or a complete lack of accessible information) regarding health insurance eligibility, payment options, and the lack of predictability of costs of services. Making this information accessible and in multiple languages is necessary for improving system navigation.

In the interim, steps should be taken to reduce the costs and complexities associated with care–for example, additional regulation and standardization of the fees charged by providers for uninsured services to bring out-of-pocket costs for patients in line with insured costs are recommended. Additionally, expanded prescription coverage for those without provincial health insurance could be a concrete step to address some of the gaps identified in our study. With the province of British Columbia expanding pharmacists’ prescribing capacities in 2023 to include some forms of contraception and medication to treat minor ailments, providing free or low-cost prescription coverage for im/migrants who do not have access to private insurance or qualify for BC PharmaCare could support increased access, particularly where this would forgo the added costs of needing to consult with a primary care physician in order to receive the prescription.

There is also a broader need for rights-based program and policy changes to address the role of ***immigration policy*** in perpetuating gaps in health insurance coverage and barriers to accessing care for those with precarious immigration status. Shifts in immigration policy towards ‘status for all’ are recommended to reduce precarity and support immigrants’ human rights more effectively [43]. These recommendations are aligned with global commitments to uphold im/migrants’ rights and the human right to health.

Amidst existing policy gaps, our findings highlighted the crucial role of ***community-based organizations*** and members for minimizing harm and supporting im/migrants’ navigation of oppressive immigration and health systems. Our findings mirror other research highlighting the essential role of community organizations in supporting im/migrant populations’ needs [44], and support calls for increased resource allocation for such efforts [23]. Given the support these organizations are already offering individuals to fill gaps in provincial information sharing, increased government funding allocated to community-based organizations which provide these vital services and fill gaps in existing systems is urgently needed.

### Strengths and limitations

As im/migrants with precarious status are under-represented and often entirely absent from population-based and epidemiological health research, a strength of our work is generating unique insights regarding lived experiences with health insurance and impacts on health and human rights for this population in BC. As sampling focused on representing a range in experiences among recently arrived im/migrant women facing precarious circumstances, findings may not represent the vast diversity of experiences of im/migrants in BC or elsewhere. To ensure rigor in our interpretation of data, we maintained a detailed study codebook; employed “member-checking” with participants; and sought analytic consensus among multiple coders. Finally, our community engaged research approach helped to ensure the relevance and accuracy of our findings to the local community and supported meaningful and trusting relationships with participants and community members.

### Conclusion

In contrast to common perceptions of Canada’s health system as ‘universal’, participant narratives highlighted the ways in which ineligibility for public health insurance coverage resulted in unmet needs for essential sexual and reproductive health care, preventive care, and curative services among im/migrant women, children, and families. Additionally, ineligibility for public health insurance resulted in negative impacts including a high economic burden, and perpetuated experiences of discrimination, invisibility, and exclusion from systems of care amongst im/migrant participants. Expanding health insurance options to cover all residents and decoupling health insurance eligibility from immigration status are recommended, alongside implementation of local-level ‘Sanctuary’ policies, which would reduce access barriers, better reflect principles at the foundation of ‘universal’ health systems and meet global commitments to uphold the right to ‘health for all’.
